# Supplementary material for: Semi-classical origin of the extreme magnetoresistance in PtSn4
Source: Nat Commun. 2024 May 29;15:4585. doi: 10.1038/s41467-024-48709-z (PMC11137119; doi:10.1038/s41467-024-48709-z)
Supplement: Supplementary file 1 — Supplementary Information [file 41467_2024_48709_MOESM1_ESM.pdf]

SUPPLEMENT - Semi-classical origin of the extreme magnetoresistance in $PtSn_4$

J. Diaz[1], K. Wang[2], J. Straquadine[1], C. Putzke[1,2], Qun Yang[3], Binghai Yan[3], S. L. Bud'ko[4], P. C. Canfield[4], P.J.W. Moll[1,2,*]

[1]Institute of Materials (IMX), Ecole Polytechnique Fédérale de Lausanne (EPFL), 1015 Lausanne, Switzerland

[2]Max Planck Institute for Structure and Dynamics of Matter, 22761 Hamburg, Germany

[3]Department of Condensed Matter Physics, Weizmann Institute of Science, Rehovot 7610001, Israel

[4]Ames Laboratory U.S. DOE and Department of Physics and Astronomy, Iowa State University, Ames, Iowa 50011, USA

S1 – Measurements on bulk crystals

FIB-machined microchannels are ideally suited to probe the physics of directional transport in ultra-pure metals. The extreme geometric factor enhances the resistance given the low resistivity of the material; and the precise channel geometry probes transport along set crystallographic directions precisely. The latter avoids the common admixture of undesired elements of the resistivity tensor due to imperfections in the current flow (e.g. microcracks) and non-ideal current injection (e.g. in manually prepared silver contacts). At the same time, FIB machining creates an amorphized / heavily disordered surface that spans into the bulk for approximately 10-20nm. Furthermore, confining the channel geometry to micron-scale bars introduces classical confinement effects and, in general, the physics of ballistic conductors. It is therefore important to check that the collapse of XMR is indeed a bulk property arising from the Fermi surface, and not induced by the microstructure fabrication.

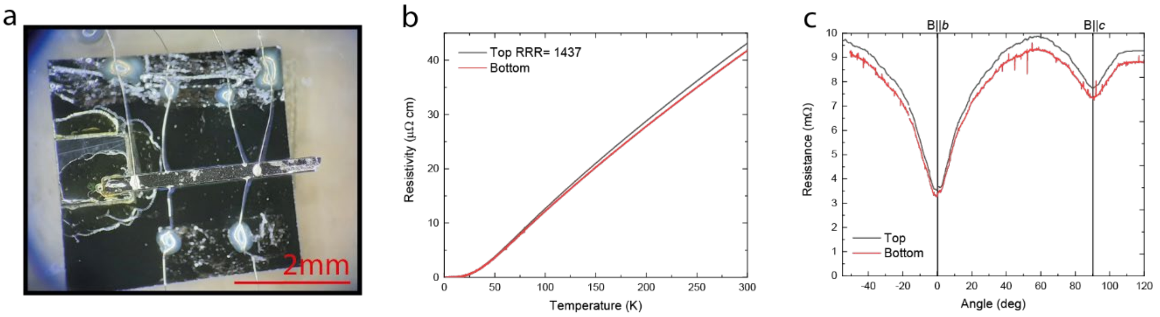

***Fig. S1** a) Bulk crystal needle manually contacted in a 6-point geometry. The 3mm long needle along **a** is free-hanging, anchored only at the left end to avoid and strain. It features two contact pairs for longitudinal transport that should be identical for homogeneous currents, referred to as "top" and "bottom". b) Resistivity vs temperature of the bulk sample. The qualitative and quantitative behavior matches well the microstructures. The difference between top and bottom is a measure of current inhomogeneity, which is a constant issue in high conductivity crystals. Their similarity evidences almost homogeneous flow in the material. c) The sample shows a pronounced minimum in resistance at 2K, 5T for fields along the b-direction.*

A long needle-like crystal was aligned and mounted with DuPont 4929 silver paint in a 6-point configuration (Fig. S1). The comparison between macro- and micro-sample reflect the above considerations well. Quantitatively, the value and temperature dependence of the resistivity is virtually the same as for the micro-structure. However, despite great care, the nominally identical top

and bottom pairs of contacts do not show the same voltage. This discrepancy is larger than the possible errors in their geometric difference, and the field-angle rotation (Fig. S1c) shows the ratio between the signals to depend on the field angle. This points to a slight deviation of the current flow from the idealized bar, a common situation in highly conductive crystals. At the same time, the residual resistivity ratio (RRR) at 1437 is larger than that of the microstructures, which usually fell slightly below 1000. This is expected to arise from semi-classical confinement of ballistic carriers.

The angle rotation in the (bc)-plane reflects the results of the microstructures well. A sharp minimum for (I||a, B||b) appears, yet not as sharp as in the microstructures. This is expected as the 3D current profile in such a configuration inevitably mixes in flow directions that experience XMR. While this captures the essential qualitative features, it also shows the difficulty of resolving the sharpness of the feature in bulk samples.

At the same time, the minimum around (I||a, B||c) is more pronounced compared to the microstructures. This points to an active role of finite size confinement in the B||c configuration for microstructured samples.

S2 – Current along the c-direction

The cylindrical Fermi surface sheet identified in the main text extends along the c-direction, for which the Fermi velocity averages to zero. In the proposed semi-classical scenario, hence a regular XMR is expected even for fields along the b-direction.

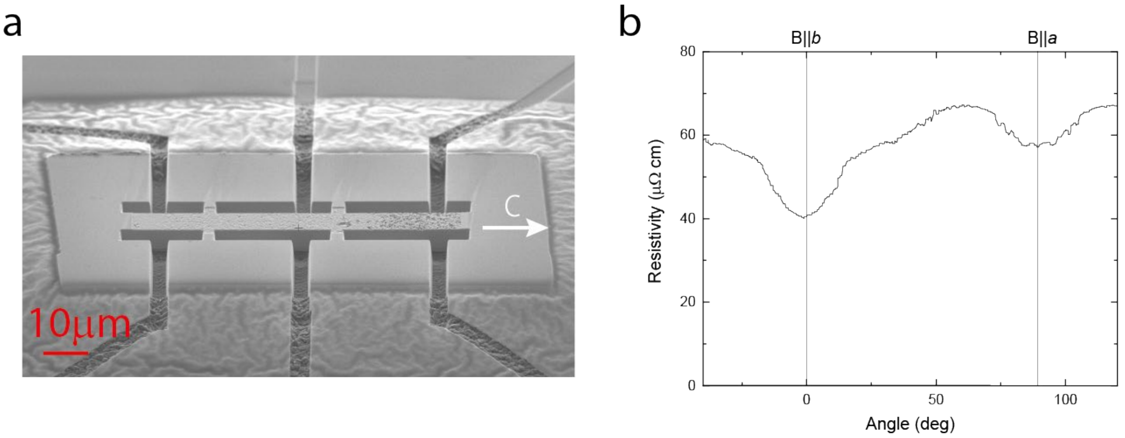

Fig. S2 *a) Crystalline microbar carved from a bulk crystal for transport along the c-direction. Two large paddles allow for homogeneous current injection, and a 6-point contact configuration is used. Indeed the signals for both channels are virtually indistinguishable. b) Angle-rotation of this sample at 2K, 14T.*

This is indeed observed (Fig. S2). For such c-direction aligned samples, only a modest anisotropy is found in the entire angle range, in particular no sharp dip appears around B||*b*.

S3 – Quantum oscillations

The long mean-free-path of the pure metal $PtSn_4$ in combination with its complex Fermi surface gives rise to a rich spectrum of quantum oscillations, which had been reported by multiple groups[1–6]. While quantum oscillations in principle allow for a tomographic reconstruction of the Fermi surface, in practice this is limited when multiple bands derived from many orbitals are present in complex crystals. These complex geometries (see main Fig. 4) yield a substantial number of locally minimal or maximal orbits, which limits the experimental feedback for ab-initio calculations to rather qualitative statements on connectivity and dimensionality.

The first and most important aspect of quantum oscillations in the context of our work is the strong evidence for the high crystalline quality of the microstructures that has not degraded by FIB-machining. In such clean metals, most commonly oscillations of magnetization, the dHvA effect, are used for Fermiology due to their favorable signal scaling in bulk crystals. Quantum oscillations in resistivity, the SdH effect, is usually much more challenging due to the low voltage signals resulting from the high conductivity of such crystals at levels of current that are compatible with the cryogenic requirements. FIB microstructuring, however, has been shown to mitigate this problem by creating long, narrow bars with favorable cross-sections that enhance the total resistance at a given resistivity. In effect, SdH oscillations at quality rivaling that of dHvA are regularly seen in FIB-microstructures, just as in $PtSn_4$ (Fig. S3).

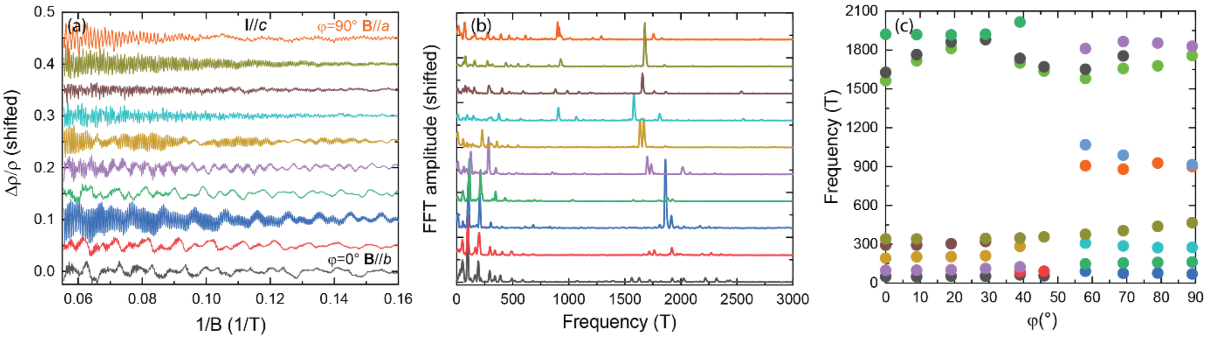

Fig. S3 Quantum oscillations a) Angle-dependent magnetoresistance at 2K after subtracting a slowly-varying polynomial. The rich oscillatory spectrum is clearly apparent in the raw data. b) The FFT analysis detects the presence of multiple sharp oscillations. c) angle-dispersion of the oscillations.

The observed oscillations are in good agreement with previous reports on bulk crystals. A broad band of 3D-like pockets in the range of 0-300T has been studied intensively and claimed to be topological[2]. The bifurcated band in the 1500T-2000T range has been partially ascribed to the circumference of the central pocket at Γ (called α-band in ref.[3]). This is a highly reasonable assignment as this is the only sheet large enough to host such large orbits. The terminated band for fields close to the a-direction (between 60°-90°) in the 900T-1000T range has been observed[3] however no Fermi surface feature matches it.

References

1. Jo, N. H. *et al.* Extremely large magnetoresistance and Kohler's rule in PdSn4: A complete study of thermodynamic, transport, and band-structure properties. *Phys. Rev. B* **96**, 165145 (2017).

2. Wang, Y. J. *et al.* Topological nature of the node-arc semimetal PtSn4 probed by de Haas-van Alphen quantum oscillations. *J. Phys. Condens. Matter* **30**, 155701 (2018).

3. Yara, T. *et al.* Small Fermi surfaces of PtSn4 and Pt3In7. *Phys. B Condens. Matter* **536**, 625–633 (2018).

4. Inamdar, M. *et al.* Quantum oscillations in ultra pure PtSn4. in *Solid State Phenomena* vol. 194 88–91 (2013).

5. Mun, E. *et al.* Magnetic field effects on transport properties of PtSn4. *Phys. Rev. B* **85**, 035135 (2012).

6. Yan, J. *et al.* The giant planar Hall effect and anisotropic magnetoresistance in Dirac node arcs semimetal PtSn4. *J. Phys. Condens. Matter* **32**, 315702 (2020).
